# Supplementary material for: cfDNA Chimerism and Somatic Mutation Testing in Early Prediction of Relapse After Allogeneic Stem Cell Transplantation for Myeloid Malignancies
Source: Cancers (Basel). 2025 Feb 13;17(4):625. doi: 10.3390/cancers17040625 (PMC11853444; doi:10.3390/cancers17040625)
Supplement: Supplementary file 1 [file cancers-17-00625-s001.zip › cancers-3405985-supplementary.pdf]

cfDNA Chimerism and Somatic Mutation Testing in Early Prediction of Relapse after Allogeneic Stem Cell Transplantation

SUPPLEMENTARY MATERIALS

SUPPLEMENTAL METHODS:

Transplant Conditioning and GvHD Prophylaxis Regimens

Patients eligible for this study received pre-transplant conditioning and GvHD prophylaxis regimens selected by the physician caring for the patient. Conditioning regimens are described by intensity (myeloablative, MA; non-myeloablative, NMA; reduced intensity, RIC) with or without rabbit antithymocyte globulin (ATG, 4-6 mg/kg in divided doses) as part of the conditioning:[1] MA consists of busulfan 130 mg/m^2 IV daily x4 on days -4 to -2; fludarabine 30 mg/m^2 IV daily x4 on days -4 to -2. NMA consists of fludarabine 30 mg/m^2 i.v. daily x5 on days -6 to -2; cyclophosphamide 14.5 mg/kg i.v. daily x2 on days -6 to -5; total body irradiation (TBI) 2 Gy x1 fraction on day -1. RIC regimens consist of: A) fludarabine 30 mg/m^2 iv daily x4 on days -5 to -2; melphalan 100 or 140 mg/m^2 iv x1 on day -2; B) melphalan 100 mg/m^2 on day -6; fludarabine 40 mg/m^2 iv daily x4 on days -5 to -2; TBI, 2 Gy x1 fraction on day -1; C) fludarabine 30 mg/m^2 IV daily x5 on days -7 to -3; busulfan 130 mg/m^2 IV daily x2 on days -4 to -3; ATG 2 mg/kg iv daily x3 on days -4 to -2.

GvHD prophylaxis was either methotrexate (MTX) or cyclophosphamide (PTCy) based, with the addition of abatacept (10 mg/m^2 IV days -1, +5, +14, +/- 28) per physician discretion: MTX-based regimen consists of methotrexate 5 mg/m^2 IV on days +1, 3, 6, 11; and tacrolimus 1 mg i.v. daily starting day -2, adjusted to achieve a therapeutic level of 5–15 ng/mL, tapered off by day 180 in absence of GvHD. PTCy regimen consists of cyclophosphamide 50 mg/kg iv days +3, +4; mycophenolate mofetil 15 mg/kg bid days +5 to +35; tacrolimus 1 mg i.v.. daily, adjusted to achieve a therapeutic level of 5–15 ng/mL, tapered off by day 180 in the absence of GvHD.

Donor Selection and Cell Source

Donors were chosen by the patient’s physician. Stem cell grafts were collected using standard techniques, and no processing of grafts (other than cryopreservation for donor management purposes and red cell depletion of ABO major incompatible bone marrow grafts) was performed. Peripheral blood stem cell (PBSC) grafts were collected using standard techniques after granulocyte colony-stimulating factor (G-CSF) mobilization with a target dose of 4-6x10^8 CD34+ cells/kg patient weight. The target for bone marrow harvesting was >3 × 10^8 nucleated cells per kilogram, but cell quantities above or below this target were infused without adjustment in number. Unrelated donor (URD) grafts were obtained through

the National Marrow Donor Program or similar registries. Per institutional practices, male sex and younger age were given priority in donor selection. Day 0 was defined as the day the cell product infusion was completed.

Post-transplant Supportive Care:

Patients were hospitalized until neutrophil engraftment, control of any infectious complications, and resolution of severe regimen-related complications. Patients were then seen in post-transplantation follow-up at least weekly through day 100 after transplantation, biweekly through day +180, and then at least monthly through 12 months. All patients received a standard (adjusted for individual patient needs) antimicrobial prophylaxis regimen starting on initiation of the transplantation conditioning regimen consisting of valacyclovir 500 mg po bid continued for at least 12 months, ciprofloxacin 500 mg po bid until neutrophil engraftment, and fluconazole 400 mg po daily through day 84 or discontinuation of corticosteroid therapy, if given. Posaconazole or voriconazole was substituted for fluconazole for patients with GVHD requiring higher doses or prolonged courses of corticosteroids. Letermovir, 480 mg daily for CMV prophylaxis commencing at transplant admission through at least day +100 (or longer per physician discretion) was administered to CMV seropositive patients. Pneumocystis carinii pneumonia (PCP) prophylaxis using trimethoprim/sulfamethoxazole, atovaquone, or dapsone was initiated after engraftment achieved and continued until completion of any GvHD prophylaxis and/or treatment.

All patients received G-CSF 5 mcg/kg/day sc rounded to vial size starting on day +5 (haploidentical donor transplantation) or day +9 (all other patients) until achieving a sustained absolute neutrophil count (ANC) of $\geq 0.5 \times 10^9$/L.

Leukocyte-depleted and irradiated blood products were given prophylactically for hemoglobin <7 gm/dL or platelet count $<10 \times 10^9$/L.

Relapse was defined as the persistence or recurrence of disease meeting standard definitions of disease relapse, documented by BM biopsy or peripheral blood (PB) sample, and requiring re-initiation of therapy or infusion of donor lymphocytes (DLI). Persistence or recurrence of minimal residual disease (MRD) based on NGS (or other technique) testing of PB or BM samples was not defined as relapse. The date of relapse was the date the BM biopsy or diagnostic PB sample confirming relapse was obtained.

Engraftment and Routine Chimerism Analysis

Neutrophil recovery *was* defined as the 1st day of 3 sequential measurements at daily intervals of a rising ANC $\geq 0.5 \times 10^9$/L and platelet engraftment as the 1st day of 3 sequential measurements at daily intervals of a rising platelet count $\geq 20 \times 10^9$/L without transfusion support during the preceding two days. Primary graft failure was defined by the lack of neutrophil engraftment by 28 days after

transplantation. Secondary graft failure was defined by initial neutrophil engraftment followed by subsequent sustained decline in the ANC to <0.5 × 10^9/L, unresponsive to growth factor therapy, with documented loss of donor CD3 chimerism, not attributed to relapse of disease, and confirmed by bone marrow analysis. $CD3^+$ donor cell chimerism analysis was performed on peripheral blood samples obtained at 4-week intervals beginning at day 28 through day 84 by PCR analysis of single tandem repeats (STR) specific to the donor and recipient in peripheral blood samples enriched for $CD3^+$ cells. Similar testing of peripheral blood cells for donor myeloid ($CD15^+$) engraftment was performed on samples enriched for $CD15^+$ cells. Patients were considered fully donor chimeric if the $CD3^+$ fractions were ≥95% donor. Post-transplant BM samples were tested for donor chimerism using $CD34^+$ cell-enriched samples. Timing of routine chimerism analysis and collection of BM samples could be modified by the physician caring for the patient.

DNA and RNA Extraction and Sequencing:

BM and PB samples were processed within 72 hours of collection. DNA and RNA from BM cells were extracted using the Maxwell® RSC 48 instruments as per the protocol provided by the manufacturer. cfDNA and cfRNA were extracted from plasma samples using Apostle MiniMax™ High Efficiency cfDNA Isolation Kit (Pleasanton, CA) following manufacturer's recommendations. The kit extracts total nucleic acid. RNA is separated by digesting DNA using DNase.

DNA Sequencing:

Similar DNA sequencing protocols were used for DNA extracted from BM cells or from plasma. The sequencing is based on KAPA HyperCap protocol from Roche (Indianapolis, IN). This workflow combines KAPA EvoPlus library preparation with KAPA HyperCap probe-based enrichment into a single workflow. The panel contains 302 hematology-related genes. The library construction is using Roche KAPA EvoPlus and KAPA HyperCapture Reagent Kits. The KAPA Universal Adapters were implemented with the KAPA Unique Dual-Indexed (UDI) Primer Mixes to remove duplicates and sequence error correction. After library amplification and cleanup, KAPA HyperChoice MAX custom probes and HyperCapture reagent kit is used for target enrichment. The final library was quantified and loaded on the NovaSeq 6000 system and run with 151x2 cycles. Sequencing data were analyzed using Dragen v3.10.8 – Somatic DNA-Seq pipeline with Unique Molecular Identifiers (UMI) analysis. The variant cell format (VCF) file generated was annotated and analyzed along following rigors protocol including inspecting binary alignment map (BAM) file for every reported mutation.

RNA Sequencing:

We used Roche KAPA RNA HyperPrep Kits for the performing RNA-seq libraries. A custom-designed targeting panel of 1,554 genes is established using KAPA HyperExplore probe design algorithm. The assay is based on hybridization/capture target enrichment using the KAPA RNA HyperPrep kit. This library preparation supports library construction from lower-input amounts and degraded samples and is compatible with mRNA capture. The final library was quantified and loaded on the NovaSeq 6000 system (Illumina, San Deigo, CA) and run with 100x2 cycles. Average read was between 80 and 100 million. Sequencing data was analyzed by Dragen v 3.10.8 – RNA-Seq pipeline using GRCh38 as reference for Fusion and GRCh37 as reference for mutation (VCF. File). Expression (TPM) data were analyzed using Salmon v 1.4.0 with GRCh38 as reference.

SUPPLEMENTAL TABLES:

Table S1. Initial diagnosis details and pre- and post-transplant cytogenetics.

Subj No.	DX	Current Status	Diagnostic Sample	Transplant Diagnostic Karyotype	Transplant Diagnostic NGS		Day Pre-Transplant Staging Obtained	Pre-transplant Staging BM Karyotype	Day 84 BM Karyotype
					Mutation	VAF			
001	AML	Expired, RRT, day 72	BM	46,XY[26]	**RUNX1 (Germline)**<br>**p.Arg250His**<br>CD36<br>p.Arg386Trp<br>SRSF2<br>p.Pro95Arg<br>BCOR<br>p.Asn820LysfsTer37<br>DNMT3a<br>p.Arg899Cys<br>IDH1<br>p.Arg132Cys<br>STAG2<br>p.Met1042ThrfsTer13<br>SETBP1<br>p.Gly870Ser<br>SMC1A<br>promoter (-2A>G)	54.13<br><br>41.33<br><br>37.19<br><br>33.94<br><br>33.83<br><br>33.62<br><br>20.63<br><br>10.82<br><br>6.85	-42	47,XY,+mar[4]/46,XY[20]	ND
002	t-MDS	Alive, day 365+	BM	46,X,t(X;1)(q13;p32), t(3;8)(p10;p10),t(3;19) (p21;q13.3),del(5)(q22),+8, 10,+mar[cp14]/46XX[6]	TP53<br>p.(N288fs)	77.7[2]	-20	47,XX,add(1)(p13),del(3)(p21p1),de l(5)(q13q33),+8,-10, -18,+mar[cp3]/46,XX[1]	45 46,X,-X,add(1)(p22), der(3;10)(q10;p10),del(5)(q13q33),+8, +0 1mar[cp2]//46,XY[22]
003	MDS	Alive, day 365+	BM	46,XY[20]	RUNX1<br>TET2	5.7<br>48.4	-22	46,XY[20]	46,XY[20]
004	AML	Expired, infection, day +357	BM	46,XX[20]	NPM1<br>ATM<br>DNMT3a<br>DNMT3a	45<br>44<br>45<br>44	-26	46,XX[20]	46,XX[20]
005	t-MDS	Alive, day 365+	BM	46,XY[20]	CHEK2<br>p. Asp390Glu<br>TP53<br>p. Arg196Ter<br>BRAF<br>p. Ile572Val<br>PPM1D<br>p. Asn431LyfsTer3<br>PPM1D<br>p.Cys478Ter<br>DNMT3A<br>p.Ile310Th<br>**DNMT3A (RNA)**<br>**p. Gln606Ter**	16.67<br><br>8.87<br><br>7.57<br><br>4.21<br><br>3.81<br><br>2.2<br><br>**14.29**	-26	46,XY[20]	46,XX[20]

006	2nd-AML	Alive, day 365+	BM	46,XY[20]	TET2<br>p.T212fs<br>TET2<br>p.N1346	50.3<br><br>48.4	-9	46,XY[20]	46,XY[20]
007	2nd-AML	Expired, relapse day 250	BM	46,XX,inv(3)(q21q26.2)[22]	SRSF2<br>p.Pro95Arg<br>NRAS<br>p.Gln61Arg<br>ASXL1<br>p.Gly927Ter	46.11<br><br>41.82<br><br>35.71	-26	46,XX,inv(3)(q21q26.2)[20]	46,XX,inv(3)(q21q26.2)[20]/46,XX[1]
008	AML	Alive, day 365+	BM	46,XY[23]	NPM1<br>p.Trp288CysfsTer12<br>GATA2<br>p.Arg362dup<br>FL3TKD<br>p.Asn676Lys<br>GATA2<br>p.Thr355GlnfsTer32<br>GATA2<br>p.Asn351Ile<br>NRAS<br>p.Gly12Asp<br>PTPN11<br>p.Asp61Tyr<br>FLT3TKD<br>p.Asp835Val<br>FLT3TKD<br>p.Asp835Tyr	55.74<br><br>40.61<br><br>16.67<br><br>14.13<br><br>13.3<br><br>9.13<br><br>3.54<br><br>2.58<br><br>2.58	-34	46,XY[20]	46,XY[21]
LB1406-009	AML	Expired, relapse day 285	BM	46,XY,t(6;9)(p22;q34)[19]/46,XY[1]	WT1<br>p.Arg370GlyfsTer17<br>NFKBIA<br>p.Glu294ProfsTer14<br>NRAS<br>p.Gln61Lys<br>WT1<br>p.Val371AlafsTer16<br>TET2<br>p.Arg1261His<br>FLT3ITD<br>p.Glu598_Tyr599insSerTyrValAspPheArgGluTyrGlu	47.62<br><br>39.55<br><br>29.03<br><br>17.18<br><br>13.04<br><br>11.59	-20	46,XY,t(6;9)(p22;q34)[1]/46,XY[20]	46,XY,t(6;9)(p22;q34)[1]/46,XY[19]
010	2nd-AML	Expired, relapse day 125	PB	46,XX[20]	CBL<br>p.Cys396Phe<br>SRSF2<br>p.Pro95Arg<br>NPM1<br>p.Trp288CysfsTer12<br>IDH2<br>p.Arg140Gln	59.15<br><br>51.17<br><br>48.35<br><br>19.12	-15	46,XX,t(14;21)(q24;q22)[3]/46,XX[19]	48,XX,+19,+21[9]/46,XX[11]
011	2nd-AML	Alive, day 365+	BM	44,XY,add(5)(q15),-7,der(9)t(1;9)(p31;q34),add(14(p11.2),dic(16;17)(p11.2;p11.2),del(20)(q11.2;q13.3)[19]/46/XY[1]	ND		-20	71,XYY,+2,-3,add(5)(q11.2),+6,-7,+8,add(9)(q34),-11,-12,+14,add(14)(p11.2),+15,der(16;17)(q10;q10),(14)(p11.2),+15,der(16;17)(q10;q10),+19,del(20)(q11.2q13.3),+21[cp3]/46,XY[5]<br>Limited Study	46,XX[20]

012	MDS	Alive, day 347	PB	46,XX[5]	SF3B1<br>p.Lys700Glu<br>**MYB (RNA)**<br>**p.Arg294Leu**	24.67<br><br>**24.65**	-30	46,XX[20]	46,XX[22]
013	MDS	Alive, day 365+	BM	46,XY[20]	TP53<br>p.Tyr236Cys	33	-38	46,XY[23]	46,XY[20]
015	AML	Alive, day 346	BM	46,XY[20]	NRAS<br>p.Gly12Asp<br>FLT3<br>p.Asp839Gly<br>FLT3<br>p.Ile836del	43<br><br>8<br><br>4	-16	46,XY,inv(9)(p12q13)c[20]	46,XX[20]
016	MDS	Died, sepsis, day 268	BM	47,XY,+8[15]/46,XY[5]	ND		-23	47,XY,+8[21]	46,XY[20]
017	CML-MBP	Alive, day 282	PB	46,XX,t(9;11)(p21;q23), t(9;22)(q34;q11.2)[20]	ASXL1<br>p.His633Ter<br>**SMARCA5 (RNA)**<br>**p.Thr704Ile**<br>**NSD1 (RNA)**<br>**p.Met1531CysfsTer43**	32.39<br><br>**27.86**<br><br>**1.04**	-17	46,XX,t(9;22)(q34;q11.2)[6]/46, XX [14]	46,XY[20]
018	2nd-AML	Alive, day 235	PB	46,XX[5]	ND		-34	46,XX[21]	46,XX[20]
019	2nd-AML	Alive, day 238	BM	46,XY,del(6)(p22.2), add(6)(p25.3)[4]/46,XY [17]	CALR<br>U2AF1<br>ASXL1<br>ASXL1	NA<br>NA<br>NA<br>NA	-29	46,XY,add(6)(p21)[20]	46,XY[20]
020	AML	Alive, day 212	BM	46,XY,del(5)(p22q35)[17]/46,XY[3]	STAG2<br>DNMT3A<br>ASXL1<br>IDH1	39.1<br>35.1<br>21.3<br>20.6	-19	46,XY,del(5)(q22q34)[1]/46,XY [19]	46,XY[20]
021	MDS	Alive, day 192	BM	46,XY,del(5)(q15q33)[19]/46,XY[1]	ETNK1<br>p.Asn244Ser<br>RUNX1<br>p.Pro261LeufsTer50<br>ASXL1<br>p.Gly642Ter<br>FLT3<br>p.Ile562Thr	41<br><br>35<br><br>4<br><br>1	-26	46,XY[11]	46,XY[20]

Table S1: Shown are transplant diagnosis, current clinical status at time of study analysis, and details regarding cytogenetic and NGS testing (if reported) of initial bone marrow (BM) or blood sample (PB) obtained to establish diagnosis. Diagnostic samples were PB cells for subjects 010, 012, 017, 018 and BM for all other subjects enrolled. Also shown are the cytogenetic results of the pre-transplant marrow staging study and the day relative to transplantation this sample was obtained. Cytogenetic analysis of the “day 84” post-transplant bone marrow sample

is also shown (the actual timing of the post-transplant bone marrow samples is shown in Table S5). Genes shown in bold text are germline mutations or RNA mutations (as noted) detected for those patients for whom this testing was performed. cfRNA sequencing was not performed on all samples and some data is missing. Subj No=subject number. Dx=transplant diagnosis. AML=acute myelogenous leukemia. MDS=myelodysplastic syndrome. RRT=regimen related toxicities. NA=Not available. ND=Not done. VAF=variant allele frequency.

Table S2. Detected mutations and VAF in study samples.

Subj No.	Pre-Transplant Staging BM NGS		Pre-Transplant cfDNA		Day 28 cfDNA NGS		Day 56 cfDNA NGS		Day 84 cfDNA NGS		Day 84 Bone Marrow NGS	
	Gene Name	VAF	Gene Name	VAF	Gene Name	VAF	Gene Name	VAF	Gene Name	VAF	Gene Name	VAF
001	**RUNX1 (Germline)**<br>**p.Arg250His**<br>DNMT3a<br>p.Arg899Cys<br>SRSF2<br>p.Pro95Arg<br>IDH1<br>p.Arg132Cys<br>**CD36 (RNA)**<br>**p.Arg386Trp**	46.34<br><br>15.85<br><br>4.66<br><br>1.3<br><br>44.88	DNMT3A<br>p.Arg882His<br>SRSF2<br>p.Pro95Arg	28.57<br><br>15.38	**RUNX1 (Germline)**<br>**p.Arg250His**<br>SMC1A<br>p.?<br>DNMT3A<br>p.Arg899Cys<br>SETBP1<br>p.Gly870Ser<br>DNMT3A<br>p.Arg882His<br>IDH1<br>p.Arg132Cys	1.37<br><br>0.23<br><br>0.21<br><br>0.1<br><br>0.05<br><br>0.04	**RUNX1 (Germline)**<br>**p.Arg250His**<br>FLT3-TKD<br>p. Asn676Ser<br>DNMT3A<br>p.Arg899Cys<br>DNMT3A<br>p.Arg882His	8.62<br><br>1.26<br><br>0.33<br><br>0.32	ND		ND	
002	TP53<br>p.Asn288ArgfsTer54<br>HNF1A<br>p.Pro479Leu	48.34<br><br>24.54	TP53<br>p.Asn288ArgfsTer54<br>HNF1A<br>p.Pro479Leu	63.57<br><br>28.71	***FANCG (Germline)***<br>***p.Glu326Ter***<br>TP53<br>p.Asn288ArgfsTer54<br>HNF1A<br>p.Pro479Leu	20.75<br><br>1.25<br><br>0.53	***FANCG (Germline)***<br>***p.Glu326Ter***<br>TP53<br>p.Asn288ArgfsTer54<br>HNF1A<br>p.Pro479Leu	12.39<br><br>4.76<br><br>1.1	***FANCG (Germline)***<br>***p.Glu326Ter***<br>TP53<br>p.Asn288ArgfsTer54<br>HNF1A<br>p.Pro479Leu	7.93<br><br>3.19<br><br>0.84	TP53<br>p.Asn288ArgfsTer54<br>HNF1A<br>p.Pro479Leu	2.5<br><br>0.51
003	BCOR<br>p.Ser336LeufsTer45<br>KMT2D<br>p.Glu766Gly	13.85<br><br>8.8	Neg		Neg		KMT2D<br>p.Glu766Gly	2.31	Neg		Neg	
004	DNMT3A<br>p.Arg882Cys	44.48	DNMT3A<br>p.Arg882Cys	44.87	DNMT3A<br>p.Arg882Cys<br>***FLNA (Germline)***<br>***p.Pro637Leu***	0.5<br><br>**37.52**	DNMT3A<br>p.Arg882Cys<br>***FLNA (Germline)***<br>***p.Pro637Leu***	0.26<br><br>34.19	***FLNA (Germline)***<br>***p.Pro637Leu***	**35.39**	DNMT3A<br>p.Arg882Cys	0.28

005	CHEK2<br>p.Asp390Glu<br>TP53<br>p.Arg196Ter<br>NOTCH3<br>p.Arg244Ter<br>PPM1D<br>p.Asn431LysfsTer3<br>PPM1D<br>p.CYS478Ter	7.53<br><br>5.2<br><br>3.95<br><br>2.18<br><br>1.66	BRAF<br>p.Ile572Val<br>TP53<br>p.Arg196Ter<br>PPM1D<br>p.Asn431LysfsTer3<br>CHEK2<br>p.Asp390Glu<br>PPM1D<br>p.Leu546ProfsTer6<br>NOTCH3<br>p.Arg244Ter<br>CARD11<br>p.Asn216Ser<br>KMT2A<br>p.Lys2434AsnfsTer22<br>PPM1D<br>p.CYS478Ter<br>PMS1<br>p.Asn502LysfsTer4<br>CDK12<br>p.Arg722Ser<br>PBRM1<br>p.Asn258MetfsTer25<br>TP53<br>p.Asn239Asp<br>KEAP1<br>p.Tyr537Asn<br>CARD11<br>p.Asp56Gly<br>KMT2C<br>p.Gly313Ter	5.97<br><br>5.53<br><br>5.13<br><br>4.76<br><br>3.23<br><br>2.79<br><br>0.76<br><br>0.7<br><br>0.67<br><br>0.58<br><br>0.56<br><br>0.52<br><br>0.35<br><br>0.21<br><br>0.18<br><br>0.11	PPM1D<br>p.CYS478Ter<br>TP53<br>p.Arg196Ter	0.85<br><br>0.34	PPM1D<br>p.CYS478Ter<br>TP53<br>p.Arg196Ter	0.42<br><br>0.22	PPM1D<br>p.Cys478Ter<br>TP53<br>p.Arg196Ter	0.32<br><br>0.37	GATA3<br>p.Ser237Glnfs<br>MAP3K14<br>p.Ter236ArgextTer?<br>TET2<br>p.Ile830AsnfsTer16<br>XRCC2<br>p.Lys267AsnfsTer30<br>KMT2A<br>p.Lys2434AsnfTer22	0.95<br><br>0.47<br><br>0.37<br><br>0.21<br><br>0.19
006	**TSHR (Germline)**<br>**p.Ile81fsTer**<br>TET2<br>p.Asn1346Ter<br>EZH2<br>p.Tyr133His<br>ASXL1<br>p.Gly646TrpfsTer12<br>TET2<br>p.Thr212AsnfsTer13	54.38<br><br>52.74<br><br>47.1<br><br>33.71<br><br>23.92	TET2<br>p.Asn1346Ter<br>**TSHR (Germline)**<br>**p.Ile81fsTer?**<br>EZH2<br>p.Tyr133His<br>ASXL1<br>p.Gly646TrpfsTer12<br>TET2<br>p.Thr212AsnfsTer13	49.13<br><br>47.96<br><br>36.36<br><br>34.0<br><br>27.4	Neg		Neg		**TSHR (Germline)**<br>**p.Ile81fsTer?**<br>TET2<br>p.Asn1346Ter<br>TET2<br>p.Thr212AsnfsTer13	0.61<br><br>0.25<br><br>0.05	ASXL1<br>p.Gly646TrpfsTer12	2.55
007	SRSF2<br>p.Pro95Arg<br>ASXL1<br>p.Gly927Ter<br>NRAS<br>p.Gln61Arg<br>MTOR<br>p.Glu506Gln<br>MTOR<br>p.Arg491Pro<br>MTOR<br>p.Arg472Thr<br>KDM6A<br>p.Thr440Arg<br>ARAF<br>p.Leu572Val	55.44<br><br>50.62<br><br>41.9<br><br>31.37<br><br>30.33<br><br>27.27<br><br>6.15<br><br>4.17	NRAS (Q61R)<br>p.Gln61Arg<br>ASXL1<br>p.Gly927Ter<br>SRSF2<br>p.Pro95Arg<br>MTOR<br>p.Glu506Gln<br>MTOR<br>p.Arg491Pro<br>MTOR<br>p.Arg472Thr<br>GNAQ<br>p.Ala93Pro<br>IRF4<br>p.Pro347Arg<br>FH<br>p.Gly424Val	49.59<br><br>48.27<br><br>45.55<br><br>40.26<br><br>38.3<br><br>36.09<br><br>4.17<br><br>4.11<br><br>2.71	ASXL1<br>p.Gly927Ter	0.66	ASXL1<br>p.Gly927Ter<br>DNMT3A<br>p.Asp531Asn<br>NRAS<br>p.Gln61Arg<br>SRSF2<br>p.Pro95Arg<br>MTOR<br>p.Glu506Gln<br>MTOR<br>p.Arg491Pro	2.35<br><br>1.21<br><br>1.01<br><br>0.74<br><br>0.88<br><br>0.81	ASXL1<br>p.Gly927Ter<br>SRSF2<br>p.Pro95Arg<br>NRAS<br>p.Gln61Arg<br>FH<br>p.Gly424Val<br>MTOR<br>p.Glu506Gln<br>MTOR<br>p.Arg491Pro<br>DNMT3A<br>p.Asp531Asn	8.29<br><br>5.92<br><br>4.46<br><br>1.85<br><br>0.55<br><br>0.31<br><br>0.27	BCL6<br>p.Gln582Glu<br>GNAS<br>p.Ala437Pro<br>ASXL1<br>p.Gly927Ter<br>SRSF2<br>p.Pro95Arg<br>GNAS<br>p.Val510Leu<br>GNAS<br>p.Ala256Pro<br>NRAS<br>p.Gln61Arg<br>MTOR<br>p.Glu506Gln<br>MTOR<br>p.Arg491Pro<br>MTOR<br>p.Arg472Thr	13.43<br><br>10.41<br><br>10.24<br><br>9.4<br><br>8.67<br><br>8.46<br><br>7.63<br><br>0.73<br><br>0.48<br><br>0.8

008	Neg		Neg		Neg		Neg		Neg		Neg	
009	NRAS<br>p.Gln61Lys<br>TET2<br>p.Arg1261His	1.54<br><br>0.28	TET2<br>p.Thr1600LysfsTer7<br>TET2<br>p.Arg1261His<br>WT1<br>p.Arg370GlyfsTer17<br>NFKBIA<br>p.Glu294ProfsTer14<br>NRAS<br>p.Gln61Lys<br>FLT3-ITD<br>p.Glu598_Tyr599insSerTyrValAspPheArgGluTyrGlu	5.78<br><br>1.38<br><br>0.83<br><br>0.6<br><br>0.39<br><br>0.23	Neg		Neg		NFKBIA<br>p.Glu294ProfsTer14<br>WT1<br>p.Arg370GlyfsTer17<br>TET2<br>p.Arg1261His<br>FLT3-ITD<br>p.Glu598_Tyr599insSerTyrValAspPheArgGluTyrGlu<br>WT1<br>p.Arg370GlyfsTer17<br>NRAS<br>p.Gln61Lys<br>WT1<br>p.Val371AlafsTer16	3.77<br><br>3.02<br><br>2.73<br><br>2.4<br><br>0.41<br><br>0.13<br><br>0.12	WT1<br>p.Arg370GlyfsTer17<br>NFKBIA<br>p.Glu294ProfsTer14<br>TET2<br>p.Arg1261His<br>FLT3-ITD<br>p.Glu598_Tyr599insSerTyrValAspPheArgGluTyrGlu	0.69<br><br>0.25<br><br>0.15<br><br>0.14
010	SRSF2<br>p.Pro95Arg<br>IDH2<br>p.Arg140Gln<br>MPL<br>p.Trp515Leu	18.93<br><br>6.61<br><br>4.31	SRSF2<br>p.Pro95Arg<br>MPL<br>p.Trp515Leu<br>IDH2<br>p. Arg140Gln	37.89<br><br>23.93<br><br>15.2	SRSF2<br>p.Pro95Arg<br>MPL<br>p.Trp515Leu<br>IDH2<br>p. Arg140Gln	1.13<br><br>0.3<br><br>0.76	SRSF2<br>p.Pro95Arg<br>IDH2<br>p. Arg140Gln	26.02<br><br>14.29	IDH2<br>p.Arg140Gln<br>SRSF2<br>p.Pro95Arg	36.11<br><br>31.04	SRSF2<br>p.Trp515Leu<br>IDH2<br>p.Arg140Gln<br>KMT2C<br>p.Ser818Leu<br>MPL<br>p.Trp515Leu<br>**BAX (RNA)**<br>**p.Lys58Ter**<br>**PI4KA (RNA)**<br>**p.Arg1941Ter**	36.08<br><br>16.24<br><br>3.87<br><br>0.54<br><br>27.02<br><br>9.5
011	TP53<br>p.Ser261ValfsTer84<br>TET2<br>p.Ile1873AsnfsTer<br>PDGFRB<br>p.Val211Ala	31.54<br><br>16.72<br><br>14.79	PDGFRB<br>p.Val211Ala<br>TET2<br>p.Ile1873AsnfsTer2<br>TP53<br>p.Ser261ValfsTer84	19.86<br><br>8.62<br><br>5.26	PDGFRB<br>p.Val211Ala	1.59	PDGFRB<br>p.Val211Ala	3.4	PDGFRB<br>p.Val211Ala<br>**TSC2 (RNA)**<br>**p.Thr1623Ala**	6.32<br><br>0	Neg	
012	SF3B1<br>p.Lys700Glu<br>AXIN1<br>p.Arg545Gln<br>AMER1<br>p.Asp97Asn<br>ASXL1<br>p.Gln561Ter<br>ASXL1<br>p.Trp583GlyfsTer120<br>**MYB (RNA)**<br>**p.Arg294Leu**	17.7<br><br>16.83<br><br>8.05<br><br>6.4<br><br>4.0<br><br>5.05	AXIN1<br>p.Arg545Gln<br>SF3B1<br>p.Lys700Glu<br>H3F3A<br>p.Ala115Gly<br>EGFR<br>p.Arg958His<br>ASXL1<br>p.Gln561Ter<br>ASXL1<br>p.Pro582LeufsTer118<br>RUNX1<br>p.Ser167ArgfsTer48<br>AMER1<br>p.Asp97Asn<br>KMT2C	34.8<br><br>33.02<br><br>17.7<br><br>3.46<br><br>3.03<br><br>3.01<br><br>2.61<br><br>2.14<br><br>1.79	H3F3A<br>p.Ala115Gly<br>KMT2C<br>p.Met959Thr<br>AMER1<br>p.Asp97Asn<br>AXIN1<br>p.Arg545Gln<br>**SF3B1 (RNA)**<br>**p.Lys700Glu**	14.73<br><br>0.37<br><br>0.22<br><br>0.22<br><br>0.36	AXIN1<br>p.Arg545Gln<br>KMT2C<br>p.Met959Thr<br>AMER1<br>p.Asp97Asn	0.34<br><br>0.25<br><br>0.001	KMT2C<br>p.Arg866Gln<br>AXIN1<br>p.Arg545Gln<br>KMT2C<br>p.Met959Thr<br>EGFR<br>p.Arg958His<br>SF3B1<br>p.Lys700Glu<br>RUNX1<br>p.Ser167ArgfsTer48<br>AMER1<br>p.Asp97Asn	1.86<br><br>0.5<br><br>0.48<br><br>0.36<br><br>0.35<br><br>0.28<br><br>0.05	KMT2B<br>p.Gly7Asp<br>SF3B1<br>p.Lys700Glu<br>AXIN1<br>p.Arg545Gln<br>RUNX1<br>p.Ser167ArgfsTer48	1.68<br><br>0.49<br><br>0.25<br><br>0.22

			p.Met959Thr<br>ASXL1<br>p.Ser577Ter<br>**MYB (RNA)**<br>**p.Arg294Leu**	1.4<br><br>22.22								
013	**MKL1 (RNA)**<br>**p.Arg362Ter**	48.78	TP53<br>p.Tyr236Cys<br>TET2<br>p.Cys1135Tyr<br>NOTCH1<br>p.Met1806Val<br>TET2<br>p.Lys722SerfsTer2<br>**ERCC3 (RNA)**<br>**p.Arg109Ter**	43.38<br><br>0.78<br><br>0.47<br><br>0.19<br><br>32.14	TP53<br>p.Tyr236Cys	0.26	TP53<br>p.Tyr236Cys<br>**ERCC3 (RNA)**<br>**p.Arg109Ter**	0.66<br><br>4.03	TP53<br>p.Tyr236Cys<br>**ERCC3 (RNA)**<br>**p.Arg109Ter**	0.37<br><br>6.94	NF2<br>p.Ala137Thr<br>**MKL1 (RNA)**<br>**p.Arg362Ter**	0.37<br><br>48.33
015	**MSH3 (RNA)**<br>**p.Glu264Ter**<br>**NRAS (RNA)**<br>**p.Gly12Asp**<br>**CREBBP (RNA)**<br>**p.Trp1472Arg**	**29.8**<br><br>**0.66**<br><br>**0.51**	**CTSA (RNA)**<br>**p.Trp135Ter**	**14.34**	CTSA p.Trp135Ter	36.07	CTSA<br>p.Trp135Ter	30.3	**CTSA (RNA)**<br>**p.Trp135Ter**	**31.2**	KMT2B<br>p.Pro1101LeufsTer81<br>DNMT3A<br>p.Arg729Trp<br>**MSH3 (RNA)**<br>**p.Glu264Ter**<br>**CREBBP (RNA)**<br>**p.Trp1472Arg**<br>**CTSA (RNA)**<br>**p.Trp135Ter**	1.25<br><br>0.7<br><br>**1.6**<br><br>**0.13**<br><br>**38.11**
016	SF3B1<br>p.Lys700Glu	33.18	SF3B1<br>p.Lys700Glu<br>TNFRSF14<br>p.Met103Leu<br>KMT2D<br>p.Arg5282Ter<br>DNMT3a<br>p.Arg326Cys<br>MAP3K1<br>p.Gln525His	43.33<br><br>1.48<br><br>0.83<br><br>0.33<br><br>0.26	SF3B1<br>p.Lys700Glu	0.98	SF3B1<br>p.Lys700Glu	0.03	SF3B1<br>p.Lys700Glu	0.06	Neg	
017	ASXL1<br>p.His633Ter	6.66	ASXL1<br>p.His633SerfsTer2<br>CEBPA<br>p.Pro188_Pro189insSer<br>DNMT3A<br>p.Tyr533Cys	12.81<br><br>5.2<br><br>0.81	***DPYD (RNA, Germline)***<br>***p.Asp949Val***	44.07	ASXL1<br>p.His633SerfsTer2<br>***DPYD (Germline)***<br>***p.Asp949Val***	0.21<br><br>32.14	***DPYD (Germline)***<br>***p.Asp949Val***	42.92	***DPYD (Germline)***<br>***p.Asp949Val***	49.52
018	**BRCA1 (Germline)**<br>**p.Cys61Gly**<br>DDX41<br>p.Arg525His<br>ALK<br>p.Glu1594GlyfsTer4<br>SRSF2<br>p.Tyr3His<br>TET2<br>p.Tyr1245Cys<br>SRSF2<br>p.Pro95His	39.74<br><br>1.82<br><br>0.97<br><br>0.8<br><br>0.51<br><br>0.29	**BRCA1 (Germline)**<br>**p.Cys61Gly**<br>ALK<br>p.Glu1594GlyfsTer4<br>ALK<br>p.Gly833Glu<br>FGFR4<br>p.Pro568GlnfsTer53<br>SF3B1<br>p.Tyr623Cys<br>SF3B1	45.28<br><br>1.46<br><br>0.44<br><br>0.35<br><br>0.34<br><br>0.21	**BRCA1 Germline)**<br>**p.Cys61Gly**<br>SF3B1<br>p.Lys700Glu	8.09<br><br>0.04	**BRCA1 (Germline)**<br>**p.Cys61Gly**	9.58	**BRCA1 (Germline)**<br>p.Cys61Gly<br>**MPO (RNA)**<br>**p.Arg569Trp**<br>**CRTC3 (RNA)**<br>**p.Arg615Ter**	8.84<br><br>**42.42**<br><br>**44.3**	ND	

			p.Lys700Glu<br>TET2<br>p.Tyr1245Cys<br>SRSF2<br>p.Pro95His	0.2<br>0.17								
019	**JAK2 (Germline)**<br>**p.Arg234His**<br>CALR<br>p.Lys385AsnfsTer47<br>U2AF1<br>p.Gln157Pro<br>ASXL1<br>p.Arg693Ter<br>GNAS<br>p.Gln870Glu<br>KRAS<br>p.Asp69_Gly75dup<br>RUNX1<br>p.Trp106Ser<br>GALNT12<br>p.His234Leu<br>TP31<br>p.Val272Met<br>GRIN2A<br>p.Ala27Val<br>**CD36 (RNA)**<br>**p.Tyr325Ter**<br>**SDHA (RNA)**<br>**p.Leu649GlufsTer4**<br>**MYB (RNA)**<br>**p.Glu308SerfsTer2**	45.2<br>39.23<br>38.39<br>34.88<br>20.25<br>20.11<br>2.04<br>0.87<br>0.6<br>0.32<br>29.58<br>18.75<br>17.78	ASXL1<br>p.Arg693Ter<br>CALR<br>p.Lys385AsnfsTer47<br>U2AF1<br>p.Gln157Pro<br>GNAS<br>p.Gln870Glu<br>KRAS<br>p.Asp69_Gly75dup<br>RUNX1<br>p.Trp106Ser	19.57<br>17.76<br>15.04<br>12.98<br>12.92<br>0.8	CALR<br>p.Lys385AsnfsTer47<br>ASXL1<br>p.Arg693Ter<br>GNAS<br>p.Gln870Glu	0.71<br>0.12<br>0.07	ASXL1<br>p.Arg693Ter<br>CALR<br>p.Lys385AsnfsTer47<br>KRAS<br>p.Asp69_Gly75dup<br>GNAS<br>p.Gln870Glu<br>U2AF1<br>p.Gln157Pro	0.76<br>0.73<br>0.71<br>0.44<br>0.26	U2AF1<br>p.Gln157Pro<br>CALR<br>p.Lys385AsnfsTer47	0.18<br>0.29	**JAK2 (Germline)**<br>**p.Arg234His**<br>ASXL1<br>p.Arg693Ter<br>**SDHA (RNA)**<br>**p.Leu649GlufsTer4**<br>**CD36 (RNA)**<br>**p.Tyr325Ter**<br>**U2AF1 (RNA)**<br>**p.Gln157Pro**<br>**CALR (RNA)**<br>**p.Lys385AsnfsTer47**	48.92<br>0.06<br>17.57<br>0.81<br>0.2<br>0.11
020	**NTRK2 (Germline)**<br>**p.Ser180Gly**<br>DMNT3A.<br>p.Arg882Cys<br>NF1<br>p.Ile679AspfsTer21<br>TET2<br>p.Tyr1295Cys<br>ASXL1<br>p.Gly646TrpfsTer12<br>SMC3<br>p.Ser127Asn	47.86<br>32.74<br>4.4<br>3.72<br>2.52<br>0.87	DNMT3A<br>p.Arg882Cys<br>NF1<br>p.Ile679AspfsTer21<br>ASXL1<br>p.Gly646TrpfsTer12<br>TET2<br>p.Tyr1295Cys<br>SMC3<br>p.Ser127Asn<br>PDGFRB<br>p.Asn292His<br>KMT2C<br>p.Gly315Val	33.87<br>4.27<br>3.64<br>2.2<br>0.4<br>0.4<br>0.23	DNMT3A<br>p.Arg882Cys	0.97	DNMT3A<br>p.Arg882Cys<br>**PTGS2 (RNA)**<br>**p.Trp309Ter**	0.37<br>47.98	DNMT3A<br>p.Arg882Cys<br>**PTGS2 (RNA)**<br>**p.Trp309Ter**	0.36<br>43.23	DNMT3A<br>p.Arg882Cys<br>**NTRK2 (Germline)**<br>**p.Ser180Gly**<br>**PTGS2 (RNA)**<br>**p.Trp309Ter**	0.37<br>0.37<br>42.38

021	**MYD88 (Germline)**<br>**p.Ala6ProfsTer39**<br>DNMT3A<br>Splice<br>+1delAACTGCAAGG)<br>DNMT3A<br>p.Phe752Leu<br>**DPYD (RNA)**<br>**p.Asp949Val**	46.09<br><br>1.02<br><br>0.47<br><br>43.53	**MYD88 (Germline)**<br>**p.Ala6ProfsTer39**<br>NF1<br>(0)<br>DNMT3A<br>(0)<br>CBL<br>p.Tyr371Asn<br>DNMT3A<br>p.Phe752Leu	44.42<br><br>1.46<br><br>1.38<br><br>1.03<br><br>1.01	***CHEK2 (Germline)***<br>***p.Ile200Thr***<br>**MYD88 (Germline)**<br>**p.Ala6ProfsTer39**	40.36<br><br>6.58	***CHEK2 (Germlline)***<br>***p.Ile200Thr***<br>**MYD88 (Germline)**<br>**p.Ala6ProfsTer39**<br>CBL<br>p.Tyr371Asn	38.87<br><br>7.1<br><br>0.11	***CHEK2 (Germline)***<br>***p.Ile200Thr***<br>**MYD88 (Germline)**<br>**p.Ala6ProfsTer39**<br>CBL<br>p.Tyr371Asn	37.24<br><br>9.05<br><br>0.11	***CHEK2 (Germline)***<br>***p.Ile200Thr***<br>**MYD88 (Germline)**<br>**p.Ala6ProfsTer39**<br>**DYPD (RNA)**<br>**p.Asp949Val**	50.88<br><br>0.14<br><br>1.07

Table S2: Shown are the detected mutated genes and associated variant allele frequencies in the pre- and post-BM samples and in analysis of cfDNA in blood samples obtained pre-transplant and at days 28, 56, and 84 after transplantation. Genes noted in bold italicized text (subjects 002, 004, 017 and 021) are presumed donor-transmitted genetic variants. Germline genes of subject origin are shown in bold text. Mutations also noted in bold text are those of genes in the RNA panel and not included in the DNA panel for analysis. Multiple mutations detected in any gene are listed as multiple distinct mutations. The timing of the pre-transplant bone marrow sample relative to transplantation is shown in Table S5. Subj No=subject number. Neg=no mutations detected in that sample. ND=a sample was not obtained at this timepoint. VAF=variant allele frequency.

Table S3. NGS analysis of samples obtained in long-term follow-up of subjects.

Subj No	Day Sample #1			Day Sample #2			Day Sample #3			Day Sample #4		
		Mutation	VAF		Gene Name	VAF		Gene Name	VAF		Gene Name	VAF
001	ND											
002	166<br>PB-cfDNA	***FANCG (Germline)***<br>***p.Glu326Ter***<br>TP53<br>p.Asn288ArgfsTer54<br>HNF1A<br>p.Pro479Leu	24.12<br><br>12.3<br><br>8.25	241<br>BM	***FANCG (Germline)***<br>***p.Glu326Ter***<br>TP53<br>p.Asn288ArgfsTer54<br>HNF1A<br>p.Pro479Leu	17.22<br><br>34.22<br><br>9.62						
003	191<br>PB-cfDNA	Neg		239<br>BM	Neg		364<br>PB-cfDNA	Neg				
004	161<br>PB-cfDNA	DNMT3a<br>p.Arg882Cys	0.06	214<br>BM	***FLNA (Germline)***<br>***p.Pro637Leu***<br>BUB1B<br>p.Gln309Ter	34.99<br><br>30.21	ND					
005	224<br>PB-cfDNA	PBMR1<br>p.Asn258MetfsTer25<br>**GATA3 (RNA)**<br>**p.Ser237GlnfsTer67**	0.0006<br><br>0.37	303<br>PB-cfDNA	TP53<br>p.Arg196Ter	0.11	367<br>PB-cfDNA	TP53<br>p.Arg196Ter	0.79			
006	211<br>PB-cfDNA	TET2<br>p.Thr212AsnfsTer13	0.15	287<br>PB-cfDNA	NONE		365<br>PB-cfDNA	NONE		ND		

007	99<br>BM	BCL6<br>p.Gln582Glu<br>GNAS<br>p.Ala437Pro<br>ASXL1<br>p.Gly927Ter<br>SRSF2<br>p.Pro95Arg<br>GNAS<br>p.Val510Leu<br>GNAS<br>p.Ala256Pro<br>NRAS<br>p.Gln61Arg<br>MTOR<br>p.Glu506Gln<br>MTOR<br>p.Arg491Pro<br>MTOR<br>p.Arg472Thr<br>IRF4<br>p.Pro347Arg<br>EP300<br>p.Ile1612Ser	13.1<br><br>9.89<br><br>45.31<br><br>44.14<br><br>12.88<br><br>10.39<br><br>43.51<br><br>28.51<br><br>26.1<br><br>25.15<br><br>4.68<br><br>3.03	125<br>BM	BCL6<br>p.Gln582Glu<br>GNAS<br>p.Ala437Pro<br>ASXL1<br>p.Gly927Ter<br>SRSF2<br>p.Pro95Arg<br>GNAS<br>p.Val510Leu<br>GNAS<br>p.Ala256Pro<br>NRAS<br>p.Gln61Arg<br>MTOR<br>p.Glu506Gln<br>MTOR<br>p.Arg491Pro<br>MTOR<br>p.Arg472Thr<br>IRF4<br>p.Pro347Arg<br>GNAQ<br>p.Ala93Pro<br>**IKBKB (RNA)**<br>**p.Asp90Glu**	21.43<br><br>20.03<br><br>43.3<br><br>48.65<br><br>18.46<br><br>18.79<br><br>51.41<br><br>19.0<br><br>18.64<br><br>18.93<br><br>7.9<br><br>8.02<br><br>15.96	175<br>BM	SRSF2<br>p.Pro95Arg<br>GNAS<br>p.Val510Leu<br>**NRAS (RNA)**<br>**p.Gln61Arg**<br>**ASXL1 (RNA)**<br>**p.Gly927Ter**	0.45<br><br>0.31<br><br>0.47<br><br>0.78	237<br>PB-cfDNA	BCL6<br>p.Gln582Glu<br>GNAS<br>p.Ala437Pro<br>ASXL1<br>p.Gly927Ter<br>SRSF2<br>p.Pro95Arg<br>GNAS<br>p.Val510Leu<br>GNAS<br>p.Ala256Pro<br>NRAS<br>p.Gln61Arg<br>LRP1B<br>p.Ile3902Met<br>**IKPKP (RNA)**<br>**p.Asp90Glu**	38.81<br><br>41.77<br><br>42.02<br><br>42.65<br><br>39.55<br><br>36.4<br><br>31.82<br><br>9.33<br><br>42.42
008	177<br>PB-cfDNA	None		261<br>BM	**NCAM1 (RNA)**<br>**p.Gln669Ter**	44.19	316<br>PB-cfDNA	**NCAM1 (RNA)**<br>**p.Gln669Ter**	31.58			

009	130<br>BM	NFKBIA.<br>P.Glu294ProfsTer14<br>TET2<br>p.Arg1261His<br>FLT3-ITD<br>p.Glu598_Tyr599insSerTy<br>rValAspPheArgGluTyrGlu<br>WT1<br>p.Arg369Gly<br>WT1<br>p.Arg370THRfsTer15<br>WT1<br>p.Arg370GlyfsTer17	9.82<br>9.29<br>8.99<br>7.5<br>7.11<br>6.74	172<br>BM	NFKBIA.<br>P.Glu294ProfsTer14<br>WT1<br>p.Arg370GlyfsTer17<br>TET2<br>p.Arg1261His<br>FLT3-ITD<br>p.Glu598_Tyr599insSerT<br>yrValAspPheArgGluTyr<br>Glu<br>WT1<br>p.Arg369Gly<br>WT1<br>p.Arg370THRfsTer15<br>WT1<br>p.Gly374AlafsTer?<br>WT1<br>p.Ala375GlyfsTer10<br>CIC<br>p.Phe1269Val<br>WT1<br>p.Arg370Gly<br>WT1<br>p.Val371AlafsTer16<br>NRAS<br>p.Gln61Lys<br>WT1<br>p.Arg380GlnfsTer5<br>WT1<br>p.Arg380GlyfsTer6<br>WT1<br>p.Val371CysfsTer14<br>FLT3<br>p.0	50.0<br>50.0<br>44.63<br>42.0<br>23.33<br>23.33<br>12.0<br>12.0<br>9.43<br>8.33<br>5.38<br>4.63<br>4.27<br>4.27<br>4.25<br>4.24	221<br>BM	NFKBIA.<br>P.Glu294ProfsTer14<br>TET2<br>p.Arg1261His<br>WT1<br>p.Arg370GlyfsTer17<br>FLT3-ITD<br>p.Glu598_Tyr599insSer<br>TyrValAspPheArgGluT<br>yrGlu<br>WT1<br>p.Arg369ThrfsTer15<br>WT1<br>p.Gly373SerfsTer11<br>WT1<br>p.Ala375GlyfsTer10<br>WT1<br>p.Arg380GlyfsTer6<br>WT1<br>p.Arg370CysfsTer14<br>WT1<br>p.Arg380GlnfsTer5<br>NRAS<br>p.Gln61Lys<br>FLT3<br>p.Val592_Flu598dup	46.41<br>44.27<br>42.24<br>39.82<br>19.89<br>16.48<br>15.79<br>5.85<br>5.71<br>5.71<br>4.54<br>1.26	ND		
010	ND											
011	348<br>PB-cfDNA	PDGFRB<br>p.Val211Ala<br>**FCGBP (RNA)**<br>**p.Lys422ThrfsTer33**	1.31<br>45.2	394<br>PB-cfDNA	PDGFRB<br>p.Val211Ala<br>**FCGBP (RNA)**<br>**p.Lys422ThrfsTer33**	0.93<br>47.98	ND					

012	167<br>BM	KMT2B<br>p.Gly7Asp<br>EGFR<br>p.Arg958His<br>RUNX1<br>p.Ser167ArgfsTer48<br>AXIN1<br>p.Arg545Gln<br>SF3B1<br>p.Lys700Glu	17.29<br><br>12.82<br><br>10.54<br><br>10.51<br><br>9.22	226<br>BM	KMT2B<br>p.Gly7Asp<br>EGFR<br>p.Arg958His<br>RUNX1<br>p.Ser167ArgfsTer48<br>AXIN1<br>p.Arg545Gln	0.22<br><br>0.16<br><br>0.14<br><br>0.13	339<br>BM	KMT2B<br>p.Gly7Asp<br>SF3B1<br>p.Lys700Glu<br>AXIN1<br>p.Arg545Gln<br>RUNX1<br>p.Ser167ArgfsTer48<br>PTPN11<br>p.Pro491Ser<br>PTPN11<br>p.Thr73Ile<br>HGF<br>p.Val484Ile<br>SETPB1<br>p.Pro382Ser<br>FANCD2<br>p.Leu1158Phe<br>KMT2C<br>p.Met959Thr	46.12<br><br>35.96<br><br>34.61<br><br>29.82<br><br>14.36<br><br>14.01<br><br>13.36<br><br>11.96<br><br>9.18<br><br>1.02	ND		
013	139<br>BM	TP53<br>p.Tyr236Cys<br>NF2<br>p.Ala137Thr<br>**MKL1 (RNA)**<br>**p.Arg362Ter**	2.76<br><br>1.52<br><br>40.0	221<br>BM	NF2<br>p.Ala137Thr<br>TP53<br>p.Tyr236Cys<br>**MKL1 (RNA)**<br>**p.Arg362Ter**	10.45<br><br>6.88<br><br>27.05	308<br>PB-cfDNA	TP53<br>p.Tyr236Cys<br>NF2<br>p.Ala137Thr<br>TET2<br>p.Cys1135Tyr	42.91<br><br>39.98<br><br>0.14	356<br>BM	TP53<br>p.Tyr236Cys<br>NF2<br>p.Ala137Thr<br>TET2<br>p.Cys1135Tyr<br>**ERCC3 (RNA)**<br>**p.Arg109Tyr**	47.7<br><br>43.91<br><br>0.76<br><br>27.08
015	158<br>BM	KMT2B<br>p.Pro1101LeufsTer81<br>**CTSA (RNA)**<br>**p.Trp135Ter**<br>**FLT3 (RNA)**<br>**p.Asp839Gly**	0.61<br><br>38.03<br><br>0.75	248<br>PB-cfDNA	DNMT3A<br>p.Arg729Trp<br>**CTSA (RNA)**<br>**p.Trp135Ter**	0.27<br><br>15	ND					
016	165<br>PB-cfDNA	**HLA-DRB1 (RNA)**<br>**p.Tyr107Ter**	13.66	ND								
017	ND											
018	ND											
019	163<br>PB-cfDNA	**JAK2 (Germline)**<br>**p.Arg234His**	51	261<br>PB-cfDNA	**JAK2 (Germline)**<br>**p.Arg234His**<br>SDHA<br>p.Leu649GlufsTer4	50.21<br><br>7.29						

020	81<br>PB-cfDNA	**NTRK2 (Germline)**<br>**p.Ser180Gly**<br>DNMT3A<br>p.Arg882Cys<br>**PTGS2 (RNA)**<br>**p.Trp309Ter**	3.17<br><br>0.08<br><br>38.85	173<br>PB-cfDNA	**NTRK2 (Germline)**<br>**p.Ser180Gly**<br>**PTGS2 (RNA)**<br>**p.Trp309Ter**	18.78<br><br>50.0	ND					
021	116<br>PB-cfDNA	***CHEK2 (Germline)***<br>***p.Ile200Thr***<br>**MYD88 (Germline)**<br>**p.Ala6ProfsTer39**	44.29<br><br>0.05	ND								

Table S3: Shown are mutations detected on analysis of peripheral blood (PB) or bone marrow (BM) samples obtained after day 84 samples obtained per physician discretion. Genes noted in bold italicized text (subjects 002, 004, 017 and 021) are presumed donor-transmitted genetic variants. Germline genes of subject origin are shown in bold text. Genes also noted in bold text are RNA assays not included in VAF analysis. The day after transplantation the sample was obtained and the sample source analyzed are listed. Subj No=subject number. Neg=No mutations detected. ND=Not done and no subsequent samples obtained during study follow-up. VAF=variant allele frequency.

Table S4. Study samples with RNA mutations identified.

Subj No	Pre-Transplant Staging BM NGS		Pre-Transplant cfRNA		Day 28 cfRNA NGS		Day 56 cfRNA NGS		Day 84 cfRNA NGS		Day 84 Bone Marrow NGS	
	Gene Name	VAF	Gene Name	VAF	Gene Name	VAF	Gene Name	VAF	Gene Name	VAF	Gene Name	VAF
001	CD36<br>p.Arg386Trp	44.88										
002												
003												
004												
005												
006												
007												
008												
009												
010											BAX (RNA)<br>p.Lys58Ter<br>PI4KA (RNA)<br>p.Arg1941Ter	27.02<br><br>9.5
011									TSC2 (RNA)<br>p.Thr1623Ala	0		
012	MYB (RNA)<br>p.Arg294Leu	5.05	MYB (RNA)<br>p.Arg294Leu	22.22	SF3B1 (RNA)<br>p.Lys700Glu	0.36						
013	MKL1 (RNA)<br>p.Arg362Ter	48.78	ERCC3 (RNA)<br>p.Arg109Ter	32.14			ERCC3 (RNA)<br>p.Arg109Ter	4.03	ERCC3 (RNA)<br>p.Arg109Ter	6.94	MKL1 (RNA)<br>p.Arg362Ter	48.33
015	MSH3 (RNA)<br>p.Glu264Ter<br>NRAS (RNA)<br>p.Gly12Asp<br>CREBBP (RNA)<br>p.Trp1472Arg	29.8<br><br>0.66<br><br>0.51	CTSA (RNA)<br>p.Trp135Ter	14.34					CTSA (RNA)<br>p.Trp135Ter	31.2	MSH3 (RNA)<br>p.Glu264Ter<br>CREBBP (RNA<br>p.Trp1472Arg<br>CTSA (RNA)<br>p.Trp135Ter	1.6<br><br>0.13<br><br>38.11
016												
017					*DPYD (RNA)*<br>*p.Asp949Val*	44.07						
018									MPO (RNA)<br>p.Arg569Trp<br>CRTC3 (RNA)<br>p.Arg615Ter	42.42<br>44.3		
019	CD36 (RNA)<br>p.Tyr325Ter<br>SDHA (RNA)<br>p.Leu649GlufsTer4<br>MYB (RNA)<br>p.Glu308SerfsTer2	29.58<br><br>18.75<br><br>17.78									SDHA (RNA)<br>p.Leu649GlufsTer4<br>CD36 (RNA)<br>p.Tyr325Ter<br>U2AF1 (RNA)<br>p.Gln157Pro	17.57<br><br>0.81<br><br>0.2

											CALR (RNA) p.Lys385AsnfsTer47	0.11
020							PTGS2 (RNA) p.Trp309Ter	47.98	PTGS2 (RNA) p.Trp309Ter	43.23	PTGS2 (RNA) p.Trp309Ter	42.38
021	DYPD (RNA) p.Asp949Val	43.53									DYPD (RNA) p.Asp949Val	1.07

Table S4: Shown are specific RNA mutations detected in RNA analysis during study period (RNA mutations also listed in Supplemental Tables S1 and S2). Subj No=subject number. VAF=variant allele frequency.

Table S5. Chimerism values.

Subj No.	Day +28				Day +56				Day +84				Day 84 Marrow	
	Day Obtained	CD3	CD15	% Donor DNA	Day Obtained	CD3	CD15	% Donor DNA	Day Obtained	CD3	CD15	% Donor DNA	Day Obtained	CD34
001	29	93	100	99	56	91	100	84	ND	ND	ND	ND	ND	ND
002	28	82	100	92	57	83	100	61	86	85	99	35	79	95
003	29	15	100	ND	55	25	100	95	82	37	100	96	61	97
004	28	87	100	97	56	86	100	72	86	91	100	92	56	98
005	33	83	100	98	57	78	100	99	85	48	100	96.5	106	96
006	29	89	100	98	56	92	100	94	ND	ND	ND	84	83	96
007	28	31	100	98	56	30	100	92	ND	ND	ND	78	64	3
008	30	100	100	99	54	100	100	98	93	100	100	97	86	98
009	30	85	100	ND	55	100	100	99	99	89	96	76	64	91
010	27	91	99	96	ND	ND	ND	50	ND	ND	ND	12	53	40
011	34	100	100	91.5	57	100	100	88	83	100	100	87	83	100
012	27	27	100	82	55	18	99	82	83	15	100	76	90	73
013	25	34	99	95	55	50	99	92	ND	ND	ND	84	74	93
015	28	74	100	ND	56	80	100	40	84	85	100	57	98	95
016	25	33	100	ND	54	69	100	94	88	65	100	89	54	88
017	29	91	100	97	60	88	100	96.5	88	88	100	92	88	94
018	29	98	100	84	54	98	100	81	90	98	100	ND	110	98
019	29	100	100	90	ND	ND	ND	92	75	100	100	90	82	100
020	21	84	100	60	55	81	100	74	69	82	100	72	69	94
021	28	89	100	86	58	72	100	79	115	93	100	78	71	99

Table S5: Shown are percent donor chimerism using STR techniques in peripheral blood samples enriched for $CD3^+$ and $CD15^+$ cells and in bone marrow samples enriched for $CD34^+$ cells. Also shown is percent donor DNA determined from NGS blood samples obtained on days

28, 56, and 84 after transplantation. Day obtained is the day after transplantation the peripheral blood and bone marrow chimerism studies were obtained. Subj No=subject number. ND=Test not performed.

Table S6. Correlation of chimerism results using NGS and STR analyses.

	Day 28		Day 56		Day 84		Day 84 BM
	CD3	CD15	CD3	CD15	CD3	CD15	CD34
NGS vs STR	0.04	-0.16	-0.07	-0.05	-0.09	0.27	0.39

Shown are Pearson's correlation coefficients comparing cfDNA of donor origin to donor $CD3^+$ and $CD15^+$ cells determined by STR analysis of sorted peripheral blood cells. Also shown is the correlation between cfDNA of donor origin to donor $CD34^+$ cells isolated from post-transplant bone marrow samples. Graphs of these correlations are shown as Supplemental Figure S1A–D.

Table S7. List of 302 genes validated and included in the DNA analysis.

No. 1-65	No. 66-130	No. 131-195	No. 196-260	No. 261-302
ABL1	CREBBP	GRIN2A	NOTCH1	SMARCA4
ABRAXAS1 (FAM175A)	CRLF2	H3-3A (H3F3A)	NOTCH2	SMARCB1
ACVR1B	CSF1R	H3C2 (HIST1H3B)	NOTCH3	SMC1A
AKT1	CSF3R	HGF	NPM1	SMC3
AKT2	CTCF	HNF1A	NRAS	SMO
AKT3	CTNNA1	HOXB13	NSD1	SOCS1
ALK	CTNNB1	HRAS	NSD2 (WHSC1)	SOX2
AMER1	CUX1	HSP90AA1	NTHL1	SOX9
ANKRD26	CXCR4	ID3	NTRK1	SPOP
APC	CYLD	IDH1	NTRK2	SRC
AR	DAXX	IDH2	NTRK3	SRSF2
ARAF	DDR2	IGF1R	PAK3	STAG2
ARID1A	DDX41	IKZF1	PALB2	STAT3
ARID1B	DICER1	IKZF3	PAX5	STAT5B
ARID2	DNM2	IL7R	PBRM1	STK11
ASXL1	DNMT3A	INHBA	PDGFRA	SUFU
ATM	DOT1L	IRF4	PDGFRB	SUZ12
ATR	EED	JAK1	PHF6	TAL1
ATRX	EGFR	JAK2	PIK3CA	TCF3
AURKA	EGLN1	JAK3	PIK3R1	TENT5C (FAM46C)

AURKB	ELANE	KAT6A	PIK3R2	TERC
AURKC	EP300	KDM5C	PIM1	TERT
AXIN1	EPAS1	KDM6A	PLCG1	TET2
AXIN2	EPCAM	KDR	PMS1	TGFBR2
B2M	EPHA3	KEAP1	PMS2	TMEM127
BAP1	EPHA5	KIT	POLD1	TNFAIP3
BARD1	ERBB2	KMT2A	POLE	TNFRSF14
BCL2	ERBB3	KMT2B	POT1	TP53
BCL2L1	ERBB4	KMT2C	PPM1D	TRAF3
BCL6	ERG	KMT2D	PPP2R1A	TSC1
BCOR	ESR1	KRAS	PRDM1	TSC2
BCORL1	ETNK1	LRP1B	PRKAR1A	TSHR
BCR	ETV6	MAP2K1	PRKDC	U2AF1
BIRC3	EXO1	MAP2K2	PRPF8	U2AF2
BLM	EZH2	MAP2K4	PRSS1	UBA1
BMPR1A	FANCA	MAP3K1	PTCH1	VHL
BRAF	FANCC	MAP3K14	PTEN	WT1
BRCA1	FANCD2	MAPK1	PTPN11	XPO1
BRCA2	FANCE	MCL1	RAC1	XRCC2
BRIP1	FANCF	MDM2	RAD21	XRCC3
BTK	FANCG	MDM4	RAD50	ZNF217
CALR	FAS	MED12	RAD51	ZRSR2
CARD11	FBXW7	MEF2B	RAD51C	

CBL	FGF4	MEN1	RAD51D	
CBLB	FGF6	MET	RAF1	
CBLC	FGFR1	MITF	RB1	
CCND1	FGFR2	MLH1	RET	
CCND3	FGFR3	MPL	RHEB	
CCNE1	FGFR4	MRE11 (MRE11A)	RHOA	
CD274 (PD-L1)	FH	MSH2	RIT1	
CD79A	FLCN	MSH3	RNF43	
CD79B	FLT3	MSH6	ROS1	
CDC73	FLT4	MTOR	RUNX1	
CDH1	FOXL2	MUTYH	SAMD9	
CDK12	FUBP1	MYC	SAMD9L	
CDK4	GALNT12	MYCL	SDHA	
CDK6	GATA1	MYCN	SDHB	
CDKN1B	GATA2	MYD88	SDHAF2	
CDKN2A	GATA3	NBN	SDHC	
CDKN2B	GEN1	NF1	SDHD	
CDKN2C	GNA11	NF2	SETBP1	
CEBPA	GNAQ	NFE2	SETD2	
CHEK1	GNAS	NFE2L2	SF3B1	
CHEK2	GNB1	NFKBIA	SMAD2	
CIC	GREM1	NKX2-1	SMAD4	

Table S8. List of 1408 genes included in the RNA analysis.

Genes Included in Analysis					
**No. 1-250**	**No. 251-500**	**No. 501-750**	**No. 751-1000**	**No. 1001-1250**	**No. 1251-1408**
ABCC3	CNBP	GAS1	MAP2	PPP2R1A	TCF12
ABI1	CNOT2	GAS5	MAP2K1	PPP2R1B	TCF3
ABL1	CNTN1	GAS7	MAP2K2	PPP2R2B	TCF7L2
ABL2	CNTRL	GATA1	MAP2K3	PPP2R4	TCL1A
ABLIM1	COG5	GATA2	MAP2K4	PPP3CA	TCL6
ACACA	COL11A1	GATA3	MAP2K5	PPP3CB	TCTA
ACE	COL1A1	GATA6	MAP2K6	PPP3CC	TEAD1
ACER1	COL1A2	GBP2	MAP2K7	PPP3R1	TEAD2
ACKR3	COL3A1	GDF6	MAP3K1	PPP3R2	TEAD3
ACSBG1	COL6A3	GFAP	MAP3K14	PPP4C	TEAD4
ACSL3	COL9A3	GHR	MAP3K6	PQLC3	TEC
ACSL6	COMMD1	GID4	MAP3K7	PRCC	TENM1
ACVR1B	COX6C	GIT2	MAPK1	PRDM1	TERF1
ACVR1C	CPNE1	GLI1	MAPK3	PRDM16	TERF2
ACVR2A	CPS1	GLI3	MAPK8	PRDM7	TERT
ADD3	CPSF6	GMPS	MAPK8IP2	PRF1	TET1
ADM	CRADD	GNA11	MAPK9	PRG2	TET2
AFF1	CREB1	GNA12	MAPRE1	PRICKLE1	TFAP2A
AFF3	CREB3L1	GNA13	MATK	PRKACA	TFDP1
AFF4	CREB3L2	GNAI1	MAX	PRKACG	TFE3
AGR3	CREBBP	GNAQ	MB21D2	PRKAR1A	TFEB
AHCYL1	CRKL	GNAS	MBNL1	PRKCA	TFG
AHI1	CRLF2	GNG4	MBTD1	PRKCB	TFPT
AHR	CRTC1	GOLGA5	MCL1	PRKCD	TFRC
AHRR	CRTC3	GOPC	MDC1	PRKCG	TGFB2
AIP	CSF1	GOSR1	MDH1	PRKDC	TGFB3

AK2	CSF1R	GOT1	MDM2	PRKG2	TGFBI
AK5	CSF3	GPC3	MDM4	PRMT1	TGFBR2
AKAP12	CSF3R	GPHN	MDS2	PRMT8	TGFBR3
AKAP6	CSNK1G2	GPR124	MEAF6	PROM1	THADA
AKAP9	CSNK2A1	GPR128	MECOM	PRRX1	THBS1
AKR1C3	CTCF	GPR34	MED12	PRRX2	THRAP3
AKT1	CTDSP2	GRB10	MEF2B	PRSS8	TIAM1
AKT2	CTLA4	GRB2	MEF2BNB-MEF2B	PSD3	TIRAP
AKT3	CTNNA1	GRHPR	MEF2C	PSEN1	TLL2
ALDH1A1	CTNNB1	GRID1	MEF2D	PSIP1	TLR4
ALDH2	CTNND2	GRIN2A	MELK	PSMD2	TLX1
ALDOC	CTRB1	GRIN2B	MEN1	PTBP1	TLX3
ALK	CTRB2	GRM1	MET	PTCH1	TMEM127
AMER1	CTSA	GRM3	METTL18	PTCRA	TMEM230
AMH	CUX1	GSK3B	METTL7B	PTEN	TMEM30A
ANGPT1	CXCL8	GSN	MFNG	PTGS2	TMPRSS2
ANKRD28	CXCR4	GSTT1	MGEA5	PTK2	TNC
ANLN	CXXC4	GTF2I	MGMT	PTK2B	TNF
APC	CYFIP2	GTSE1	MIB1	PTK7	TNFAIP3
APH1A	CYLD	H2AFX	MIPOL1	PTPN11	TNFRSF10B
APLP2	CYP1B1	H3F3A	MIR1260B	PTPN2	TNFRSF10D
APOD	CYP2C19	HAS2	MIR4321	PTPN6	TNFRSF11A
AR	DAB2IP	HDAC1	MIR4683	PTPRA	TNFRSF14
ARAF	DACH1	HDAC2	MIR4758	PTPRK	TNFRSF17
ARFRP1	DACH2	HDAC3	MIR6515	PTPRO	TNFRSF6B
ARHGAP20	DAXX	HDAC4	MIR6752	PTPRR	TOP1
ARHGAP26	DCLK2	HDAC5	MIR6765	PTTG1	TOP2A
ARHGEF12	DCN	HDAC6	MIR6795	PVT1	TOP2B
ARHGEF7	DDB2	HDAC7	MIR6857	RABEP1	TP53

ARID1A	DDIT3	HECW1	MIR6894	RAC1	TP53BP1
ARID2	DDR2	HEPH	MITF	RAC2	TP63
ARIH2	DDX10	HERPUD1	MKI67	RAC3	TP73
ARNT	DDX20	HES1	MKL1	RAD21	TPD52L2
ARRDC4	DDX39B	HES5	MKL2	RAD50	TPM3
ASMTL	DDX3X	HEY1	MLF1	RAD51	TPM4
ASPH	DDX5	HGF	MLH1	RAD51B	TPO
ASPSCR1	DDX6	HHEX	MLLT1	RAD51C	TPR
ASTN2	DEK	HIF1A	MLLT10	RAD51D	TRAF2
ASXL1	DGKB	HIP1	MLLT11	RAD52	TRAF3
ATF1	DGKI	HIPK1	MLLT3	RAF1	TRAF5
ATF3	DGKZ	HIPK2	MLLT4	RALGDS	TRHDE
ATG13	DICER1	HIST1H1C	MLLT6	RANBP17	TRIM24
ATG5	DIRAS3	HIST1H1D	MMP7	RANBP2	TRIM27
ATIC	DIS3L2	HIST1H1E	MMP9	RAP1GDS1	TRIM33
ATL1	DKK1	HIST1H2AC	MN1	RARA	TRIP11
ATM	DKK2	HIST1H2AG	MNAT1	RASAL1	TRPS1
ATP1B4	DKK4	HIST1H2AL	MNX1	RASGEF1A	TSC1
ATP6V1G2-DDX39B	DLEC1	HIST1H2AM	MPL	RASGRF1	TSC2
ATP8A2	DLL1	HIST1H2BC	MRE11A	RASGRF2	TSHR
ATR	DLL3	HIST1H2BJ	MSH2	RASGRP1	TTK
ATRNL1	DLL4	HIST1H2BK	MSH3	RB1	TTL
ATRX	DMRT1	HIST1H2BO	MSH6	RBM15	TUSC3
AURKA	DMRTA2	HIST1H3B	MSI2	RBM6	TYK2
AURKB	DNAJB1	HIST1H4I	MSN	RCHY1	TYMS
AUTS2	DNM1	HLF	MTCP1	RCOR1	U2AF1
AXIN1	DNM2	HMGA1	MTOR	RCSD1	U2AF2
AXL	DNM3	HMGA2	MTUS2	RECQL4	UBE2B
BACH1	DNMT1	HMGB1	MUC1	REEP3	UBE2C

BACH2	DNMT3A	HMGN2P46	MUTYH	RELA	UFC1
BAG4	DOCK1	HNF1A	MYB	RELN	UFM1
BAIAP2L1	DOT1L	HNRNPA2B1	MYBL1	RERG	USP16
BAP1	DPM1	HOOK3	MYC	RET	USP42
BARD1	DPYD	HOXA10	MYCL	RGS7	USP5
BAX	DST	HOXA11	MYCN	RHBDF2	USP6
BAZ2A	DTX1	HOXA13	MYD88	RHOA	USP7
BCAS3	DTX4	HOXA3	MYH11	RHOD	VCAM1
BCAS4	DUSP2	HOXA9	MYH9	RHOH	VEGFA
BCL10	DUSP22	HOXC11	MYO18A	RICTOR	VEGFC
BCL11A	DUSP26	HOXC13	MYO1F	RLTPR	VGLL3
BCL11B	DUSP9	HOXD11	NAB2	RMI2	VHL
BCL2	DUX2	HOXD13	NACA	RNF213	VTI1A
BCL2A1	DUX4	HOXD9	NAPA	RNF43	WASF2
BCL2L1	DUX4L2	HRAS	NAV3	ROBO1	WDFY3
BCL2L2	DUX4L4	HSP90AA1	NBEAP1	ROBO2	WDR1
BCL3	E2F1	HSP90AB1	NBN	ROS1	WDR18
BCL6	EBF1	HSPA1A	NBR1	RPA3	WDR70
BCL7A	ECT2L	HSPA1B	NCAM1	RPL22	WDR90
BCL9	EDIL3	HSPA2	NCKIPSD	RPN1	WEE1
BCOR	EDNRB	HSPA4	NCOA1	RPN2	WHSC1
BCORL1	EED	HSPA5	NCOA2	RPS21	WHSC1L1
BCR	EEFSEC	HTRA1	NCOA3	RPS6KA1	WIF1
BDNF	EGF	HUWE1	NCOA4	RPS6KA2	WISP3
BHLHE22	EGFR	IBSP	NCOR2	RPS6KA3	WNT10A
BICC1	EGR1	ICAM1	NCSTN	RPTOR	WNT10B
BIN1	EGR2	ICK	NDC80	RREB1	WNT11
BIRC3	EGR3	ID1	NDE1	RRM1	WNT16
BIRC6	EGR4	ID3	NDRG1	RRM2B	WNT2B
BIVM-ERCC5	EIF4A2	ID4	NDUFAF1	RTEL1	WNT3

BLM	EIF4E	IDH1	NEDD4	RTEL1-TNFRSF6B	WNT4
BMP4	ELF4	IDH2	NEURL1	RTN3	WNT5B
BMPR1A	ELK4	IFNG	NF1	RUNX1	WNT6
BRAF	ELL	IFRD1	NF2	RUNX1T1	WNT7B
BRCA1	ELN	IGF1	NFATC1	RUNX2	WNT8B
BRCA2	ELOVL2	IGF1R	NFATC2	RYR3	WRN
BRD1	ELP2	IGFBP2	NFE2L2	S1PR2	WSB1
BRD3	EML1	IGFBP3	NFIB	SARNP	WT1
BRD4	EML4	IKBKB	NFKB1	SBDS	WWOX
BRIP1	ENPP2	IKBKE	NFKB2	SCN8A	WWTR1
BRSK1	EP300	IKZF1	NFKBIA	SDC4	XBP1
BRWD3	EP400	IKZF2	NGF	SDHA	XIAP
BTBD18	EPC1	IKZF3	NGFR	SDHAF2	XKR3
BTG1	EPCAM	IL12RB2	NIN	SDHB	XPA
BTG2	EPHA10	IL13	NIPBL	SDHC	XPC
BTK	EPHA2	IL13RA2	NKX2-1	SDHD	XPO1
BTLA	EPHA3	IL15	NKX2-5	SEC31A	XRCC6
BUB1B	EPHA5	IL1B	NOD1	2-Sep	YAP1
C10orf55	EPHA7	IL1R1	NODAL	5-Sep	YPEL5
C11orf1	EPHB1	IL1RAP	NONO	6-Sep	YTHDF2
C11orf30	EPHB6	IL2	NOS3	9-Sep	YWHAE
C11orf54	EPO	IL21R	NOTCH1	SERP2	YY1AP1
C11orf95	EPOR	IL2RA	NOTCH2	SERPINE1	ZBTB16
C2CD2L	EPS15	IL3	NOTCH3	SERPINF1	ZC3H7A
C2orf44	ERBB2	IL6	NOTCH4	SET	ZC3H7B
C3orf27	ERBB3	IL7R	NPM1	SETBP1	ZFP64
CACNA1F	ERBB4	INHBA	NPM2	SETD2	ZFPM2
CACNA1G	ERC1	INPP4A	NR3C1	SETD7	ZFYVE19
CACNA2D3	ERCC1	INPP4B	NR4A3	SF3B1	ZIC2

CAD	ERCC2	INPP5A	NR6A1	SFPQ	ZMIZ1
CALR	ERCC3	INPP5D	NRAS	SFRP2	ZMYM2
CAMK2A	ERCC4	IQCG	NSD1	SFRP4	ZMYM3
CAMK2B	ERCC5	IRF1	NT5C2	SGK1	ZMYND11
CAMK2G	ERCC6	IRF2BP2	NTF3	SGPP2	ZNF207
CAMTA1	ERG	IRF4	NTF4	SH2D5	ZNF217
CANT1	ERLIN2	IRF8	NTRK1	SH3BP1	ZNF24
CAPRIN1	ESR1	IRS1	NTRK2	SH3D19	ZNF331
CAPZB	ETS1	IRS2	NTRK3	SH3GL1	ZNF384
CARD11	ETS2	IRS4	NUMA1	SH3GL2	ZNF444
CARM1	ETV1	ITGA5	NUP107	SHC1	ZNF521
CARS	ETV4	ITGA7	NUP214	SHC2	ZNF585B
CASC5	ETV5	ITGA8	NUP93	SIK3	ZNF687
CASP3	ETV6	ITGAV	NUP98	SIN3A	ZNF703
CASP7	EWSR1	ITGB3	NUTM1	SIRT1	ZRSR2
CASP8	EXOSC6	ITK	NUTM2A	SKP2	
CAV1	EXT1	ITPKA	NUTM2B	SLC1A2	
CBFA2T3	EXT2	JAG2	OFD1	SLC34A2	
CBFB	EYA1	JAK1	OLIG1	SLC45A3	
CBL	EYA2	JAK2	OLIG2	SLC7A5	
CBLB	EZH2	JAK3	OLR1	SLCO1B3	
CBLC	EZR	JARID2	OMD	SLX4	
CCAR2	FAF1	JAZF1	P2RY8	SMAD2	
CCDC28A	FAM127C	JUN	PAFAH1B2	SMAD3	
CCDC6	FAM19A2	KALRN	PAG1	SMAD4	
CCDC88C	FAM19A5	KANK1	PAK1	SMAD6	
CCK	FAM46C	KAT2B	PAK3	SMAP1	
CCL2	FAM64A	KAT6A	PAK6	SMARCA1	
CCNA2	FANCA	KAT6B	PAK7	SMARCA4	
CCNB1IP1	FANCB	KCNB1	PALB2	SMARCA5	

CCNB3	FANCC	KDM1A	PAPPA	SMARCB1	
CCND1	FANCD2	KDM2B	PASK	SMC1A	
CCND2	FANCE	KDM4C	PATZ1	SMC3	
CCND3	FANCF	KDM5A	PAX3	SMO	
CCNE1	FANCG	KDM5C	PAX5	SNAPC3	
CCNG1	FANCI	KDM6A	PAX7	SNCG	
CCT6B	FANCL	KDR	PAX8	SNHG5	
CD19	FANCM	KDSR	PBRM1	SNW1	
CD22	FAS	KEAP1	PBX1	SNX29	
CD274	FASLG	KIAA0232	PC	SNX9	
CD28	FBN2	KIAA1524	PCBP1	SOCS1	
CD36	FBXO11	KIAA1549	PCLO	SOCS2	
CD44	FBXO31	KIAA1598	PCM1	SOCS3	
CD58	FBXW7	KIF5B	PCNA	SOD2	
CD70	FCGBP	KIT	PCNA-AS1	SORBS2	
CD74	FCGR2B	KLF4	PCSK7	SORT1	
CD79A	FCRL4	KLHL6	PDCD1	SOS1	
CD79B	FEN1	KLK2	PDCD11	SOX10	
CD8A	FEV	KLK7	PDCD1LG2	SOX11	
CDC14A	FGF1	KMT2A	PDE4DIP	SOX2	
CDC14B	FGF10	KMT2B	PDGFA	SP1	
CDC25A	FGF13	KMT2C	PDGFB	SP3	
CDC25C	FGF14	KMT2D	PDGFD	SPECC1	
CDC42	FGF19	KPNB1	PDGFRA	SPEN	
CDC73	FGF2	KRAS	PDGFRB	SPOP	
CDH1	FGF23	KSR1	PDK1	SPP1	
CDH11	FGF3	KTN1	PEG3	SPRY2	
CDK1	FGF4	LAMA1	PER1	SPRY4	
CDK12	FGF6	LAMA5	PFDN5	SPTAN1	
CDK2	FGF8	LAMP2	PHB	SPTBN1	

CDK4	FGF9	LASP1	PHF1	SQSTM1	
CDK5RAP2	FGFR1	LCK	PHF23	SRC	
CDK6	FGFR1OP	LCP1	PHF6	SRF	
CDK7	FGFR1OP2	LEF1	PHOX2B	SRGAP3	
CDK8	FGFR2	LEFTY2	PI4KA	SRRM3	
CDK9	FGFR3	LFNG	PICALM	SRSF2	
CDKL5	FGFR4	LGALS3	PIK3CA	SRSF3	
CDKN1A	FH	LGR5	PIK3CB	SS18	
CDKN1B	FHIT	LHFP	PIK3CD	SS18L1	
CDKN1C	FHL2	LHX2	PIK3CG	SSBP2	
CDKN2A	FIGF	LHX4	PIK3R1	SSX1	
CDKN2B	FIP1L1	LIFR	PIK3R2	SSX2	
CDKN2C	FLCN	LINC00598	PIM1	SSX2B	
CDKN2D	FLI1	LINC00982	PKM	SSX4	
CDX1	FLNA	LINGO2	PLA2G2A	SSX4B	
CDX2	FLNC	LMBRD1	PLA2G5	ST6GAL1	
CEBPA	FLT1	LMO1	PLAG1	STAG2	
CEBPB	FLT3	LMO2	PLAT	STAT1	
CEBPD	FLT3LG	LMO7	PLAU	STAT3	
CEBPE	FLT4	LNP1	PLCB1	STAT4	
CENPF	FLYWCH1	LOX	PLCB4	STAT5A	
CENPU	FNBP1	LPAR1	PLCG1	STAT5B	
CEP170B	FOS	LPP	PLCG2	STAT6	
CEP57	FOSB	LPXN	PLEKHM2	STIL	
CEP85L	FOSL1	LRIG3	PML	STK11	
CHCHD7	FOXL2	LRMP	PMS1	STL	
CHD2	FOXO1	LRP1B	PMS2	STRN	
CHD6	FOXO3	LRP5	POFUT1	STX5	
CHEK1	FOXO4	LRPPRC	POLD1	STYK1	
CHEK2	FOXP1	LRRC37B	POLD4	SUFU	

CHIC2	FRK	LRRC59	POLR2H	SUGP2	
CHL1	FRMPD4	LRRC7	POM121	SULF1	
CHMP2B	FRS2	LRRK2	POMGNT1	SUV39H2	
CHN1	FRYL	LTBP1	POSTN	SUZ12	
CHST11	FSTL3	LYL1	POT1	SYK	
CHUK	FUS	LYN	POU2AF1	SYP	
CIC	FUT1	MACROD1	POU5F1	TACC1	
CIITA	FZD10	MAD2L1	PPAP2B	TACC2	
CIRH1A	FZD2	MADD	PPARG	TACC3	
CIT	FZD3	MAF	PPARGC1A	TAF1	
CKB	FZD6	MAFB	PPFIA2	TAF15	
CKS1B	FZD7	MAGED1	PPFIBP1	TAL1	
CLP1	FZD8	MAGEE1	PPM1D	TAL2	
CLTA	GAB1	MALAT1	PPP1CB	TAOK1	
CLTC	GABRG2	MALT1	PPP1R13B	TBL1XR1	
CLTCL1	GADD45B	MAML1	PPP1R13L	TBX15	
CMKLR1	GANAB	MAML2	PPP2CB	TCEA1	

SUPPLEMENTAL FIGURES

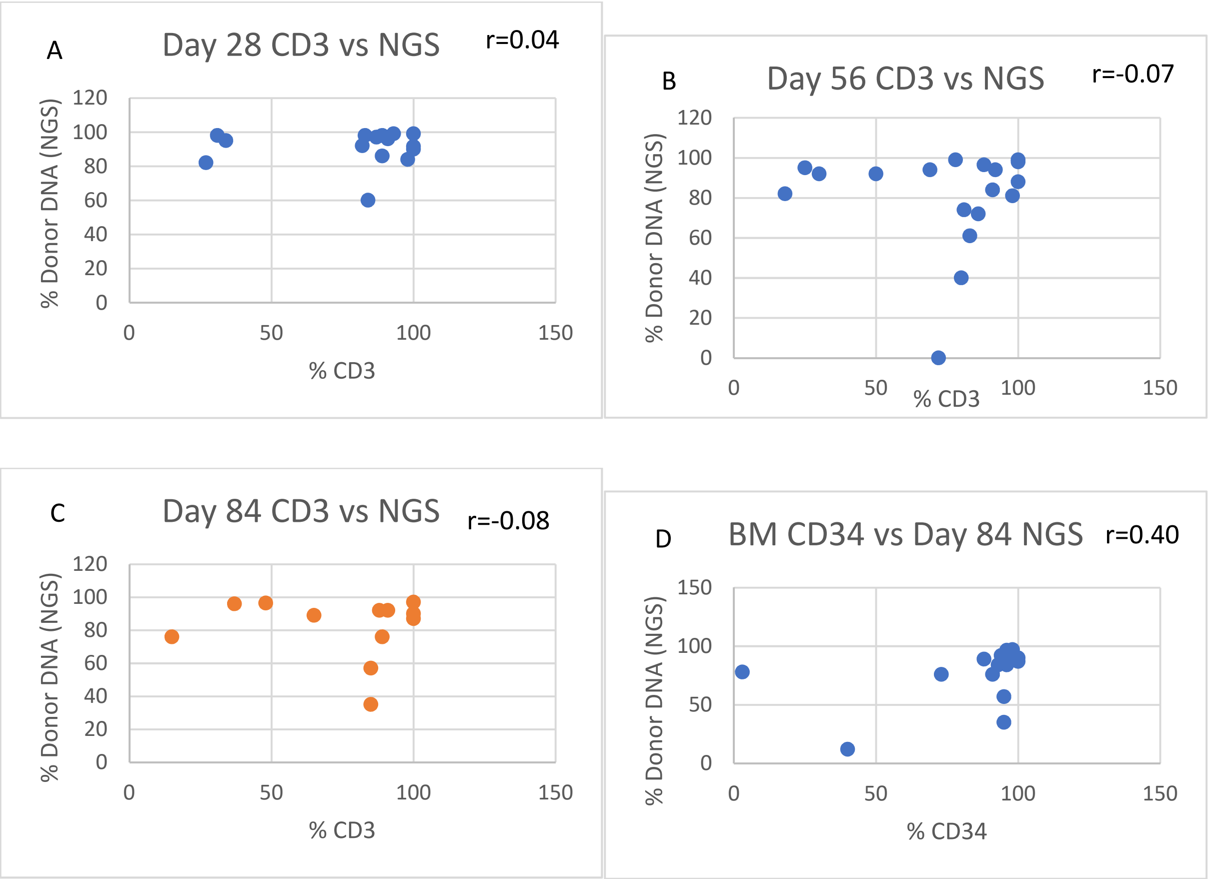

Figure Legends:

Figure S1. Shown are the correlation between chimerism tests by technique. Figure S1A shows the correlation of donor $CD3^+$ cell chimerism with cfDNA chimerism for samples obtained at day 28. Figure S1B shows the correlation of donor $CD3^+$ cell chimerism with cfDNA chimerism for samples obtained at day 56. Figure S1C shows the correlation of donor $CD3^+$ cell chimerism with cfDNA chimerism for samples obtained at day 84. Figure S1D shows the correlation of donor $CD34^+$ cell chimerism measured in post-transplant bone marrow samples with the cfDNA chimerism tested on day 84.

SUPPLEMENT REFERENCES

1 Bacigalupo A, Ballen K, Rizzo D, Giralt S, Lazarus H, Ho V, Apperley J, Slavin S, Pasquini M, Sandmaier BM et al. Defining the intensity of conditioning regimens: working definitions. *Biol Blood Marrow Transplant*. 2009;15(12):1628-33.

2 Khoury JD, Solary E, Abla O, Akkari Y, Alaggio R, Apperley JF, Bejar R, Berti E, Busque L, Chan JKC, et al. The 5th edition of the World Health Organization Classification of Haematolymphoid Tumours: Myeloid and Histiocytic/Dendritic Neoplasms. *Leukemia*. 2022;36(7):1703-1719.
